# Editorial: Gastrointestinal cancer immunotherapy: from drug resistance mechanisms to overcoming strategies

**DOI:** 10.3389/fimmu.2023.1230591

**Published:** 2023-06-07

**Authors:** Tao Shi, Hanbing Wang, Jia Wei, Jinyan Wang, Yibo Fan, Chunlei Zheng, Xiaofang Che

**Affiliations:** ^1^ Department of Oncology, Nanjing Drum Tower Hospital, Affiliated Hospital of Medical School, Nanjing University, Nanjing, China; ^2^ Department of Immunology, Basic School of Medicine, China Medical University, Shenyang, China; ^3^ Department of Gastrointestinal Medical Oncology, The University of Texas MD Anderson Cancer Center, Houston, TX, United States; ^4^ Department of Oncology, Shanghai Electric Power Hospital, Shanghai, China; ^5^ Department of Medical Oncology, The First Hospital of China Medical University, Shenyang, China; ^6^ Key Laboratory of Anticancer Drugs and Biotherapy of Liaoning Province, The First Hospital of China Medical University, Shenyang, China; ^7^ Clinical Cancer Research Center of Shenyang, the First Hospital of China Medical University, Shenyang, China

**Keywords:** gastrointestinal cancer, tumor immunotherapy, resistance, tumor microenvironment, immune checkpoint blockade

Gastrointestinal (GI) cancers have long been considered as highly heterogeneous and intractable, with high rates of morbidity and mortality globally (1). Despite the important breakthroughs and clinical success of cancer immunotherapies such as immune checkpoint blockade (ICB) therapy in some cancer types like melanoma (2), the overall response rate (ORR) of ICB therapy in the non-selective GI patients is still not satisfactory (3). About 70-80% of GI patients displayed primary resistance to ICB therapy, while some patients subsequently developed immunotherapy resistance during the treatment process (3). Both tumor-intrinsic factors, such as driver gene mutations or oncogenic pathway activation, and tumor-extrinsic factors, such as the suppressive tumor immune microenvironment (TIME), contribute to the complex drug resistance mechanisms in GI cancers. Thus, current studies aim to develop efficient overcoming strategies to improve treatment responses of immunotherapies (4). In this Research Topic, with the efforts of five guest editors, 15 articles consisting of 6 reviews, 7 original researches and 2 case reports were collected, providing a deep understanding and new comprehensive insight of immunotherapy resistance mechanisms and potential overcoming strategies in GI cancers, including esophageal cancer (EC), gastric cancer (GC), colorectal cancer (CRC) and hepatocellular carcinoma (HCC).

EC is among the deadliest malignancies due to its late-stage diagnosis and escalating worldwide incidence (5). Besides conventional therapies, immunotherapy, represented by ICB, has gained promise in treating patients with EC. To offer an objective and integrated view of research navigations to promote future advances in ICB, Yang and Wang systemically combed the publication trends and research highlights of PD-1/PD-L1 blockade therapy in EC treatment for the past ten years *via* visualized bibliometric methods. As publication characteristics were displayed varying from countries and time points in the article, the authors pointed out that current research hotspots are focused on neoadjuvant immunotherapy and biomarkers development for esophageal cancer, emphasizing the significance of developing effective biomarkers. Furthermore, Fang et al. reviewed the progress and limitations in immunotherapeutic interventions across-the-aboard, involving ICB, adoptive CAR-T cells and cancer vaccines. Since drug resistance is a crucial threat to satisfactory clinical benefits, the authors discussed resistance mechanisms from two aspects, intrinsic and acquired, and proposed that countermeasures addressing immunotherapy resistance require promising predictive biomarkers and multidisciplinary combination therapies. To find out the immunoregulatory factors related to ICB resistance, Deng et al. compared the transcriptome data of immune cells in the peripheral blood of esophageal squamous carcinoma (ESCC) patients with different responses to PD-1 blockade. They demonstrated that immune checkpoint expression was upregulated in the ICB-sensitive group and identified several genes expression (MT2A, MT1X and MT1E) correlated with ICB resistance. On the other hand, Jin et al. constructed a pipeline, ELISE (Ensemble Learning for Immunotherapeutic Response Evaluation), which incorporates ensemble deep learning and self-attention approaches for accurately predicting responses of patients with esophageal adenocarcinoma (EAC) to ICB therapy. This model based on multi-discipline techniques sheds light on exploiting robust predicting tools to promote efficacies of immunotherapies in EC and other cancers.

GC is another common GI cancer worldwide with high incidence and mortality rates and poor prognosis. Despite immunotherapy (anti-PD-1/PD-L1, programmed cell death protein 1/programmed cell death protein ligand 1) has been approved in advanced GC, the medium overall survival time is still fewer than 24 months (6). Multiple mechanisms, including the aberrant gene/pathway variations of GC cells and the inhibitory immune components in gastric TIME, may contribute to the poor response of ICB therapy. Song et al. constructed a metastasis-related epithelial-mesenchymal transition (EMT) signature (MEMTS) based on differentially expressed genes (DEGs) and EMT gene set from The Cancer Genome Atlas (TCGA) cohort and the Asian Cancer Research Group (ACRG) cohort. They found that high MEMTS predicted poor prognosis and poor response to ICB in GC with an AUC curve of 0.896. Similarly, another bioinformatic analysis based on bulk RNA-seq and single-cell RNA-seq data by Song et al. found that patients with high VCAN expression tended to be resistant to not only adjuvant chemotherapy and adjuvant chemoradiotherapy, but also immunotherapy. Therefore, both MEMTS and VCAN could serve as potential biomarkers for immunotherapy in GC patients. Besides, as for the immune-inhibitory factors in gastric TIME, the review article provided by Liu et al. highlighted the concept of ‘tumor immune tolerance’, which transforms the TIME from tumor-suppressive to tumor-promoting as the tumor progresses. They summarized that the metabolic and phenotypic changes of both innate immune cells (such as tumor-associated macrophages, neutrophils, and mast cells) and adaptive immune cells (mostly CD4^+^ and CD8^+^ T cells) could induce tumor immune tolerance, which subsequently results in the resistance of GC immunotherapy. Moreover, another review article by Kudo-Saito et al. specifically focused on the myeloid villains (including myeloid-derived suppressor cells (MDSCs), regulatory DCs (DCregs), mesenchymal stromal/stem cells (MSCs), macrophages, neutrophils, mast cells and basophils) within the TIME, which all contribute to the tumor immune suppression through different approaches, and can be targeted to improve the clinical outcomes of ICB therapy in GI cancers.

CRC is another major type of GI cancers with a high morbidity rate and poor prognosis (7). Although ICB therapy has achieved important progress in deficient mismatch repair (dMMR)/microsatellite instability-high (MSI-H) colorectal CRC, the majority of CRC patients (approximately 85%) with proficient mismatch repair (pMMR)/microsatellite stability status still respond poorly to immunotherapies (8). In this Research Topic, two reviews provided by Shan et al. and Ding et al. discussed the role and function of immunosuppressive cells (Tregs, TAMs and MDSCs), cytokines (TGF-β, VEGF, IL-4, IL-10, etc.), immune checkpoints, intestinal microbiota and nutrients within microenvironment of CRC that all bring immunotherapy resistance, and summarized the current circumstances of clinical trials that estimate the effects of immunotherapy drugs on CRC patients. As for the impact of tumor-intrinsic factors, Liu et al. identified epigenetic-related gene mutations (Epigenetic_Mut) in 18.35% of MSS-CRC patients from TCGA database and local cohorts. Epigenetic_Mut was also associated with increased infiltration of anti-tumor immune cells and favorable clinical outcomes in MSS-CRC patients receiving PD-1 blockade therapy, indicating that Epigenetic_Mut could be a potential biomarker for ICB therapy in CRC. Also worth noting in this Research Topic are two CRC cases that have opposite treatment outcomes with anti-PD-1 therapy. Although MSI-H status is considered as a favorable biomarker for immunotherapy, Zhang et al. reported a case of LS-associated CRC patient with MSI-H status who failed to benefit from anti-PD-1 therapy. The authors discussed that driver gene mutations like *KRAS* or *PTEN* mutations might be the potential reasons for the poor response of ICB therapy. Interestingly, another case reported by Yang et al. described an advanced MSS/pMMR mCRC patient who had a complete response (CR) after triple-combined therapy (PD-1 inhibitor, radiotherapy and granulocyte-macrophage colony-stimulating factor (GM-CSF)) with progression-free survival (PFS) for more than 2 years so far, thus highlighting the potential value of this triple-combination immunotherapy strategy for MSS/pMMR mCRC patients.

HCC is the most prevalent pathological type of primary liver cancer with dismal prognoses. With the approval of ICB-based therapies as standards of care (9), it is necessary to have an in-depth understanding of the complex TIME which impacts the efficacies of immunotherapy. The establishment of next-generation sequencing methods endows the possibility of analyzing cellular components in the TME and heterogeneous molecular features of HCC (10). Xu et al. conducted an integrated assessment of transcriptome sequencing, DNA mutation and clinical information in several HCC database-derived cohorts. They discovered that upregulated Wnt/β-catenin signaling signatures are potential predictors for worse-prognosis and ICB-insensitivity, which was linked with poor CD8^+^ T cell infiltration. Though this WNT-based subtyping still requires *in vitro* or *in vivo* experimental validation, it is of great clinical significance. Meanwhile, because HCC majorly forms under chronic liver inflammation, Yu et al. gave a panoramic view of two crucial components of TIME, tumor-derived exosomes and tumor-associated macrophages, in HCC tumorigenesis and progression. As exosomes play an indispensable role in macrophage polarization, the authors proposed targeting exosomes as a prospective branch of immunotherapy for HCC. Nevertheless, more investigations are needed before clinical applications.

In summary, the 15 articles in this Research Topic explore or discuss the potential drug resistance mechanisms of immunotherapies for GI cancers from different aspects, and provide possible strategies targeting both tumor cells and TIME to improve the treatment efficacies. Unfortunately, the progress of immunotherapy in pancreatic cancer, the recognized immunotherapy-resistant tumor, is not covered in this Research Topic, which is worth further exploring in the future. More research and efforts are required to achieve successful applications of immunotherapy on GI cancers in the future.

## Author contributions

TS, HW, and JWe drafted the manuscript. JWa, YF, CZ and XC reviewed and corrected the manuscript. All authors contributed to the article and approved the submitted version.
